# The Impact of Intraoperative CO_2_ Pneumoperitoneum Pressure in Gastrointestinal Surgery: A Systematic Review

**DOI:** 10.1097/SLE.0000000000001325

**Published:** 2025-02-26

**Authors:** Roy Mahapatra, Matthew Fok, Nicola Manu, Maria Cameron, Aimee Johnson, Aaron Kler, Hayley Fowler, Rachael Clifford, Dale Vimalachandran

**Affiliations:** * Department of Colorectal Surgery, Countess of Chester NHS Foundation Trust, Chester; † Institute of Systems, Molecular and Integrative Biology, University of Liverpool, Liverpool, UK

**Keywords:** low-pressure laparoscopy, systematic review, gastrointestinal surgery, pneumoperitoneum

## Abstract

**Introduction:**

Pneumoperitoneum is widely used in gastrointestinal surgery, particularly for laparoscopic or robotic procedures, with suggested advantages associated with low pressure. While existing data predominantly focuses on laparoscopic cholecystectomy, the assessment of intra-abdominal pressures in other gastrointestinal surgeries remains unexplored.

**Methods:**

This study conducted an electronic literature search for randomized control trials comparing low-pressure pneumoperitoneum to standard or high-pressure counterparts.

**Results:**

Out of 26 articles meeting inclusion criteria, encompassing 2077 patients, 15 demonstrated positive associations with low-pressure pneumoperitoneum. No significant difference in postoperative pain was found in the remaining papers. Methodological variations, diverse outcome reporting, and a prevalent high risk of bias precluded meta-analysis.

**Conclusions:**

The study highlights substantial outcome variability, urging cautious interpretation of aggregated results. Despite positive associations in specific cases, insufficient evidence was found to support the superiority of low-pressure pneumoperitoneum. The study recommends future research employing validated patient-reported outcome measures and standardized reporting to help guide the development of evidence-based guidelines and optimize patient care in abdominal surgeries.

Pneumoperitoneum is used in gastrointestinal surgery to create a potential space between the peritoneum and viscera to allow instrumentation for laparoscopic or robotic surgical procedures.^1^ Adoption of minimally invasive surgical techniques has produced putative evidence demonstrating improved surgical outcomes.^1–4^ Reported benefits include decreased blood loss, smaller surgical incisions, reduced postoperative pain, reduced length of hospital stay, earlier return of gut motility, earlier return to normal activities, and improved cosmesis.^5–9^ Minimally invasive surgery is becoming the dominant surgical paradigm in terms of surgical approach.

There is currently no standardized guidance within the UK or international literature specifying the optimal intraoperative pneumoperitoneum pressures, which should be used in gastrointestinal (GI) surgery. In addition, there is no standardized definition of low-pressure, standard (control) pressure, or high-pressure pneumoperitoneum. There is putative evidence to suggest that lower intraoperative pneumoperitoneal pressures produce reduced intraoperative complication rates and improved postoperative patient outcomes.

Pneumoperitoneum can produce adverse effects for the patient. There are significant cardiovascular and respiratory physiological adaptations which occur following the induction of pneumoperitoneum.^10,11^ Increased intra-abdominal pressure (IAP) can potentially reduce perfusion of intra-abdominal organs; venous preload; functional residual capacity (FRC); and paradoxically increase postoperative pain.^10,12–16^ The lack of standardized guidance on optimal intraoperative pneumoperitoneum pressures leaves this potentially crucial variable to the discretion of the operating surgeon.

There is evidence suggesting that reducing the intraoperative pneumoperitoneal pressure can reduce postoperative pain and the associated use of opioid analgesics.^17–19^ There is grade 1 evidence demonstrating these benefits for patients undergoing laparoscopic cholecystectomy (LC). The potential benefits for other minimally invasive GI procedures have not been assessed. Importantly, the comparability of studies has been difficult because there are no set definitions or standardized protocols for low-pressure laparoscopy. This systematic review aims to collate and assess the current evidence on whether lower pneumoperitoneum pressures can make a significant difference in outcomes in GI surgery. It also aims to set minimal reporting outcomes and standardized protocols for future such studies.

## METHODS

### Literature Search Strategy

**FIGURE 1:**
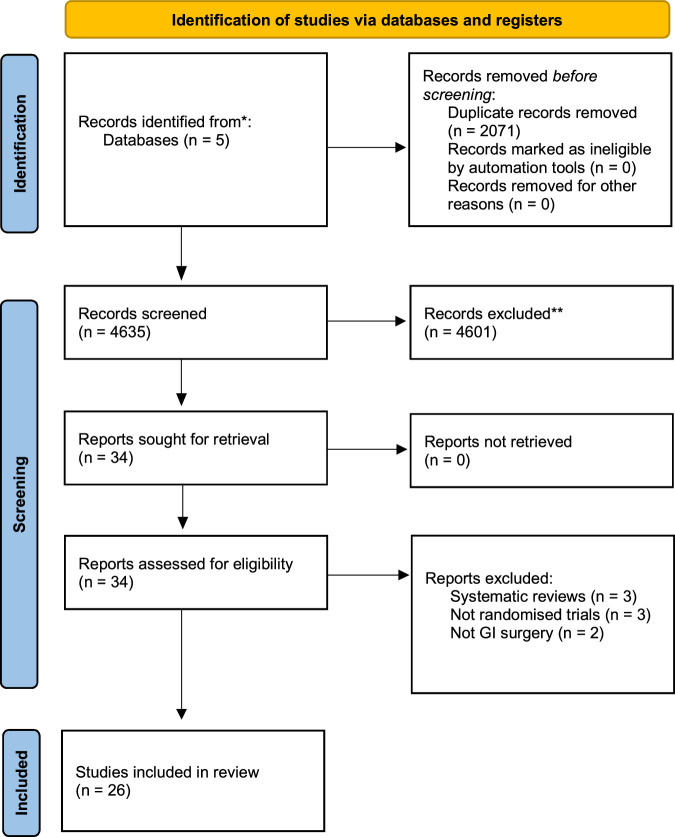
PRISMA flowchart of included studies.

A comprehensive literature search was conducted according to the Preferred Reporting Items for Systematic Reviews and Meta-Analyses (PRISMA) guidelines. A search was performed on PubMed/Medline, Scopus, Ovid, Cochrane, and Google Scholar in December 2020. Search terms included laparoscopy, laparoscopic surgery, low impact laparoscopy, low-pressure pneumoperitoneum, low-pressure pneumoperitoneum, ultra-low pneumoperitoneum pressure low-pressure laparoscopy, standard pressure pneumoperitoneum, normal pressure pneumoperitoneum. We excluded procedures that included a thoracic component. All search terms were combined with Boolean operators and searched with MeSH terms to ensure maximal sensitivity. Titles and abstracts were screened using the inclusion criteria. Full-text article reference lists were searched for any further articles that were suitable for inclusion (Fig. 1). The search process was undertaken by 2 independent investigators with discrepancies resolved by consensus with a third independent investigator (M.F.). This study was registered on the Prospero database (CRD42023411189).

The literature search identified 4635 potential studies following the removal of duplicates. Subsequent to screening of the titles and abstracts, 4601 papers were excluded. Thirty-four studies for which the full text was obtained and reviewed. Of these papers the reference list was searched to look for any potential papers to include but did not reveal any further papers. Any queries regarding the inclusion of a paper into the systematic review were resolved through consensus.

### Methodological Quality Assessment of Included Studies

A qualitative assessment of the bias of the included studies was performed using the revised tool for Risk of Bias (RoB 2 Tool).^20^ The quality of the included studies were rated by 2 reviewers and discrepancies were resolved by consensus.

### Data Extraction and Measured Outcomes

Data extraction was performed on an online spreadsheet by 2 reviewers. The primary outcome was visual analog scale (VAS) pain scores and the secondary outcomes included immune and biochemical serological markers, intraoperative measured anesthetic values, patient reported outcomes, and length of stay.

### Statistical Analysis

The planned synthesis of data included standard descriptive statistics (reported as means with 95% CI or median with an interquartile range where appropriate) to summarize demographic and operative data of recruited patients from all eligible studies. A meta-analysis of postoperative pain in low-pressure compared with normal and high-pressure pneumoperitoneum was planned to be performed using standardized mean difference (SMD) as summary statistics for the raw data extracted from included studies. The Mantel-Haenzel test was the random-effect model used to determine study variation, χ^2^ Tests to study heterogeneity and the *I* ^2^ statistic to estimate the proportion of total variation across studies due to heterogeneity rather than chance. A cutoff threshold of 40% was chosen and values exceeding this were considered to signify substantial heterogeneity. However, due to a high risk of bias shown from the methodological quality assessment of the included studies using the RoB2 tool, it was determined that conducting a meta-analysis was inappropriate and, therefore, not undertaken.

## RESULTS

A total of 26 papers met the inclusion criteria^21–46^ (Fig. 1), which incorporated a total of 2077 patients. There was a predominance of females included in the study (626 male patients; however, this variable was not reported in 4 studies, Table 1). Age was reported inconsistently and varied widely (Table 1). Of the 26 papers included in the study, the majority (n = 22) reported outcomes in patients undergoing LC. Three reported outcomes in laparoscopic/robotic colorectal surgery and 1 in bariatric surgery. Baseline demographics for all studies are displayed in Table 1.

**TABLE 1:** Baseline Demographics of Included Studies

References	No. participants	Male	Mean age	Surgery	Control pressure, (mm Hg)	Intervention pressure, (mm Hg)	No. ports	Access	Local anesthetic	Neuromuscular blockage	Intraoperative analgesia	Postoperative analgesia
Albers et al^21^	178	128	68.7	Colorectal resections	12	8	NA	NA	NA	Deep in low pressure and moderate in control (unspecified)	NA	NA
Albers et al^22^	30	20	67.4	Colorectal resections and 1 rectopexy	12 or 16	8	NA	NA	NA	Rocuronium at induction	NA	NA
Barczynski and Herman^23^	148	19	48	Elective cholecystectomy	12	7	4	Veress	NA	Pancuronium at induction	Pethidine, midazolam, and paracetamol, fentanyl	I.V. ketoprofen
Bhattacharjee et al^2,24^	80	NA	36.6	Elective cholecystectomy	14	9-10	4	Veress	Bupivacaine	Atracurium and vecuronium at induction	Fentanyl	Intravenous diclofenac sodium 6 hourly in the first 12 hours. Oral analgesics (Paracetamol 325 mg + Ibuprofen 400 mg) were given 8 hourly. Rescue analgesia injection fentanyl
Bolat and Kacmaz^25^	70	57	52.1	Elective cholecystectomy	14-16	8-10	4	Veress	NA	Rocuronium continued intraoperatively	Intravenous fentanyl	Dicofenac if VAS >4 and then IM tramadol
Caesar et al^26^	50	0	40.8	Roux-en-Y gastric bypass	18	12	5	NA	Remifentanil	Atracurium at induction	Ketobemidon, clonidine	NA
Celarier et al^27^	138	63	66	Right or left colectomy	12-15	5-7	5	NA	2% naropeine	Cisatracurium at induction and then continuous infusion for deep NMB	Ketamine and lidnocaine, morphine, ibuprofen, and paracetamol	IV morphine, paracetamol, and ibuprofen. Tramadol as rescue if the VAS was >3
Celik et al^28^	60	0	66	Cholecystectomy	12 or 14	8	4	Veress	NA	NA	NA	Pethidine, diclofenac IM PRN
Chang et al^29^	150	NA	44.2	Elective cholecystectomy	12-14	6-8 and 9-11	4	NA	NA	NA	NA	Diclofenac and PCA at request
Chok et al^30^	40	16	47.4	Elective cholecystectomy	12	7	4	Open hassan	Bupivacaine	NA	Fentanyl	Oral dologesic and diclofenac
Dey and Malik^31^	100	31	44.9	Elective cholecystectomy	13-15	10-12	4	Veress	Bupivacaine	NA	NA	NA
Esmat et al^32^	109	29	47.2	Elective cholecystectomy	14	10	4	Veress	NA	NA	NA	Ketoprofen, pethidine
Gin et al^33^	100	22	48.2	Elective cholecystectomy	12-15	8	4	Open hassan	Ropivacaine/Bupivacaine with adrenaline	Rocuronium, atracurium or vercuronium at induction and intraoperatively	Paracetamol, fentanyl, parecoxib	Paracetamol, ibuprofen, parecoxib, oxycodone
Joshipura et al^34^	26	15	57.5	Elective cholecystectomy	12	8	4	NA	Lignocaine 2%	NA	NA	NA
Kandil and Hefnawy^35^	100	38	42.4	Elective or urgent cholecystectomy	14	8 or 10 or 12	4	NA	NA	NA	NA	Ketorolac, pethindine
Kanwer et al^36^	60	NA	NA	Elective cholecystectomy	12-14	7-10	4	NA	NA	NA	NA	Diclofenac
Koc et al^37^	53	9	47.1	Elective cholecystectomy	15	10	NA	Veress	NA	NA	NA	Diclofenac
Moro et al^38^	80	12	53.5	Elective cholecystectomy	14	10	NA	NA	Ropivacaine	Rocuronium at induction	Remifentanil, ketoprofen	IV morphine, ketoprofen, dipyrone
Nasajiyan et al^39^	50	50	43.8	Urgent cholecystectomy	14-15	7-9	NA	NA	NA	Cisatracurium at induction	Midazolam, fentanyl	Apotel at recovery and at 6 hours, NSAIDs suppositories PRN
Neogi et al^40^	82	7	38.7	Cholecystectomy	14	7	4	NA	NA	Atracurium at induction	Remifentanil	Fentanyl
Perrakis et al^41^	40	10	56.8	Elective cholecystectomy	15	8	4	Veress	NA	Atracurium at induction	Lornoxicam, pethidine	Paracetamol and codeine
Sandhu et al^42^	140	27	54.6	Elective cholecystectomy	14	7	3	Open hassan	NA	NA	NA	Nalbuphine IV
Sarli et al^43^	90	24	48.5	Elective Cholecystectomy	13	9	4	NA	NA	Atracurium at induction	Fentanyl	Ketoprofen
Singla et al^44^	100	32	52.2	Elective cholecystectomy	12-14	7-8	4	NA	NA	Atracurium at induction and PRN intraoperatively	Fentanyl	Dicofenac IV 8 hourly
Vijayaraghavan et al^45^	43	17	42.3	Elective cholecystectomy	12	8	4	Veress	NA	Vecuronium at induction and intraoperatively	Diazepam night before the surgery, IM morphine 60 minutes before surgery	IV morphine PCA
Yasir et al^46^	100	NA	NA	Elective cholecystectomy	14	8	NA	NA	NA	NA	NA	NA

Elective cholecystectomy—day case cholecystectomy, urgent cholecystectomy—cholecystectomy for complicated gallstone disease, cholecystectomy—the reason for cholecystectomy is unspecified.

Definitions of low pressure and control pneumoperitoneum varied between the studies (5 to 12 and 12 to 18 mm Hg, respectively). Substantial heterogeneity was observed with respect to preoperative and intraoperative analgesia use. Surgical techniques used to gain access to the abdominal cavity varied from closed access technique (Veress needle) to open Hassan technique and “not reported.” The number of ports used to gain access to the abdominal cavity ranged from 3 to 5 and was inconsistently reported. Postoperative analgesia regimes were also inconsistently reported and varied substantially from nonopioid analgesia to strong opioid analgesia with some patients requiring IV opioid patient-controlled analgesia. There was also variation in the dose and drug type used when administrating local anesthetic to the port sites and for neuromuscular blockage, and in some studies was not reported.

In 15 of the 26 papers, there is a positive association between lower pneumoperitoneal pressures and patient outcomes. These outcomes were significantly less shoulder abdominal and overall pain; improved intraoperative pO_2_ levels; decreased postoperative analgesic consumption; and improved postoperative immune—and biochemical serological markers (Table 2). Conversely, 11 studies reported no significant difference between low-pressure and standard-pressure pneumoperitoneum with respect to VAS pain scores, analgesic consumption, shoulder tip pain, and QoR-40 score markers (Table 2). A single study found significantly increased pain on forced coughing in patients treated with low-pressure pneumoperitoneum.^38^

**TABLE 2:** Clinical Outcomes—The Colored Visual Objects Represent Improved Measured Outcome in Low-Pressure Pneumoperitoneum (Green Circle With Plus), No Difference Between Low Pressure and Standard Pressure Pneumoperitoneum (Red Circle With Minus) or No Difference in Primary Outcome Between Low Pressure and Standard Pressure But Differences Found in Secondary Outcomes (Yellow Circle With Exclamation Mark)

References	Outcome measurements	Outcomes	Visual outcome representation
Albers et al^21^	Quality of recovery 40 questionnaire	Quality of recovery was significantly higher in LP with significantly less 30-day infectious complications	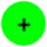
Albers et al^22^	Peritoneal perfusion measured with indocyanine green	Improved perfusion of the parietal peritoneum with LP	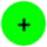
Barczynski and Herman^23^	VAS of overall pain, standard QoL questionnaire, incidence of shoulder tip pain, analgesic consumption of ketoprofen	Significantly improved VAS pain scores, incidence of shoulder tip pain, QoL scores, and reduced analgesic use with LP	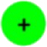
Bhattacharjee et al^24^	VAS of abdominal and shoulder tip pain, surgeon satisfaction score	Significantly lower shoulder tip pain in LP at all time points but only at 4 hours in abdominal pain. No difference in surgeon satisfaction scores	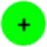
Bolat and Kacmaz^25^	VAS of abdominal and shoulder tip pain, biochemical serum measurements	Significantly improved shoulder VAS pain scores at 6 hours and 12 hours, no difference in abdominal VAS pain scores in LP	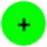
Caesar et al^26^	VAS of abdominal and shoulder tip pain, surgeon assessment of access	No difference in VAS for pain scores, significantly more difficult for surgical access in LP	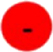
Celarier et al^27^	Length of stay, overall VAS of pain, requirement for analgesia	Significantly reduced length of stay, VAS of pain, and reduced analgesic consumption in LP	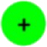
Celik et al^28^	Overall VAS pain, analgesic consumption	No difference in VAS pain scores and analgesic consumption, significant difference in operation times in LP	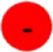
Chang et al^29^	Overall VAS pain score	No difference in overall VAS of pain between groups	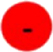
Chok et al^30^	Overall VAS pain, analgesic consumption	No difference in overall VAS of pain, or analgesic consumption, between groups	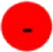
Dey and Malik^31^	Incidence of shoulder tip pain	No significant difference in the incidence of shoulder tip pain, between groups	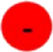
Esmat et al^32^	Incidence and VAS of shoulder tip pain, analgesic usage	Significantly reduced incidence and VAS (at 6, 12, and 24 h) of shoulder tip pain with LP	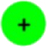
Gin et al^33^	Overall VAS pain, analgesic consumption	No difference in VAS of pain. Significantly reduced fentanyl consumption in recovery	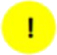
Joshipura et al^34^	Overall VAS of pain, intraoperative anesthetic values, length of stay, and analgesic consumption	Significantly improved intraoperative pO_2_ level, postoperative pain, analgesic requirement, pulmonary function, and hospital stay with LP	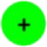
Kandil and Hefnawy^35^	Incidence and VAS of shoulder tip pain	Significantly improved incidence and VAS of shoulder tip pain with LP	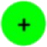
Kanwer et al^36^	Incidence of shoulder tip pain	No significant difference in the incidence of shoulder tip pain, significantly improved VAS with LP but only at 12 hours	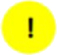
Koc et al^37^	McGill Pain Questionnaire and overall VAS of pain	No significant differences between groups	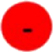
Moro et al^38^	Quality of recovery 40 questionnaire	No significant difference in the QoR-40 score. Significantly more pain with LP during forced coughing at 4, 8, and 12 hours	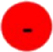
Nasajiyan et al^39^	VAS of nausea and vomiting, incidence of shoulder tip pain	Significantly reduced incidence of shoulder tip pain with LP, no difference in VAS of nausea and vomiting	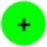
Neogi et al^40^	Serum liver enzymes postoperative, and surgeon comfort level	Significantly improved hepatic enzymes and significantly worse surgeon comfort level with LP	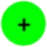
Perrakis et al^41^	VAS of abdominal pain and analgesic consumption, the incidence of shoulder tip pain, and nausea or vomiting	No significant differences in postoperative pain scores, analgesic consumption, and the incidence of nausea, vomiting, and shoulder pain between groups	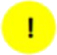
Sandhu et al^42^	Operative time, analgesic consumption, length of stay, VAS of overall pain, incidence of shoulder and back pain	No significant difference between groups	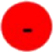
Sarli et al^43^	Incidence and VAS of shoulder tip pain	Significantly improved VAS pain scores and incidence of shoulder tip pain with LP	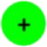
Singla et al^44^	Analgesic consumption and overall VAS pain scores	Significantly less analgesic consumption at 3-4 hours and 9-12 hours with LP. No difference in the incidence of nausea or vomiting	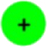
Vijayaraghavan et al^45^	Analgesic consumption and overall VAS pain scores, serum biochemical markers, PEFR, and surgeon comfort levels	Significantly reduced analgesic consumption, and overall VAS of pain at all time points, significantly reduced surgeon comfort level with LP	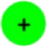
Yasir et al^46^	Incidence and VAS of shoulder tip pain	Significantly reduced incidence of shoulder tip pain and VAS of shoulder tip pain at 4 hours only with LP	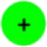

Fifteen studies reported surgical complications, although this was reported heterogeneously. Fourteen of the 15 studies reported no differences in surgical complications either intraoperatively or postoperatively. A single study reported that low-pressure pneumoperitoneum was associated with a significantly decreased rate of surgical site infection compared with control.^21^ The same study reported that in 25% of patients the surgeon requested the pressure to be increased to aid visualization.

The surgeon’s comfort level and the surgeon’s assessment of visibility was reported in 4 studies.^24,26,40,45^ Of these studies, 3 of the studies reported that the surgeon’s comfort level and the surgeon’s assessment of visibility was significantly reduced in patients treated with low pressure pneumoperitoneum.

**FIGURE 2:**
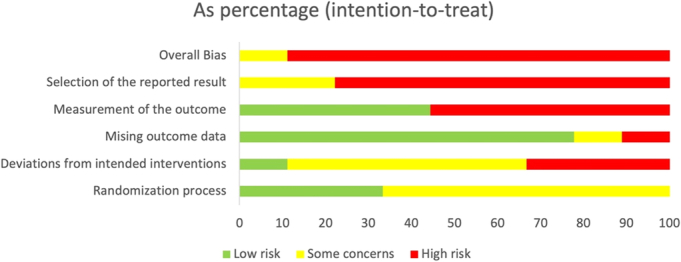
RoB 2 assessment of bias of included studies.

The risk of bias assessment of the included studies demonstrated that the majority of studies were at high risk of bias when assessing their methodological rigor and reporting quality (Fig. 2). The overall risk of bias in the studies was classed as either some concern or high concern.

## DISCUSSION

This review has demonstrated that the majority of studies assessing low-pressure pneumoperitoneum have been performed in LC. A significant finding of this systematic review is that the variability of results across studies is high. Approximately two-thirds of the reviewed literature describe significant benefits with low-pressure pneumoperitoneum, and the remaining third reported no difference (Table 2). Previous systematic reviews have endeavored to amalgamate and meta-analyse (both pairwise and network) these findings, with a majority suggesting that the pooled results favor low-pressure pneumoperitoneum.^18,19,47^ However, it is important to temper these findings with a nuanced assessment of the available evidence and consider whether meta-analysis is an appropriate tool of analysis within the context of high-risk bias.^48^ Our study has shown that the current evidence exhibits multiple limitations which encompass factors such as high susceptibility to bias, insufficient statistical power, and methodological deficiencies. It is important to emphasize that these observations are not intended as a critique of the research efforts but rather as an acknowledgment of the challenges inherent in conducting clinical trials in this complex field. However, with this in mind, this meta-analysis of these studies should be interpreted with the upmost caution and may not reflect the true evidence.

Within this study, of the 26 included studies the majority were in LC, with a combination of urgent, emergency, and elective cases. Three studies assessed major colorectal surgery and 1 study assessed bariatric surgery. It is important to recognize that the type of surgery impacts greatly on postoperative pain because of the duration of surgery, the extent of tissue dissection, and blood loss.^49^ However, importantly pain may not be the only worthwhile measurement to assess. This is reflected in a Cochrane paper on the benefits of low-pressure pneumoperitoneum, which highlights that pain scores are unvalidated surrogate outcomes for pain in people undergoing laparoscopic cholecystectomy, and several Cochrane systematic reviews have demonstrated that pain scores can be decreased with no clinical implications in people undergoing laparoscopic cholecystectomy.^47^ Patient reported outcomes (PROM’s) would make a more meaningful comparison which could be assessed with the quality of recovery (Questionnaire QoR-40). More objective measures may also point to different biochemical and immune responses patients have in response to variable pneumoperitoneal pressures. Albers was able to visualize significantly better perfusion to the peritoneum with low-pressure pneumoperitoneum, as well as reduced surgical site hypoxia and inflammation markers and circulating damage-associated molecular patterns.^22^ Other improved biochemical markers have been seen with improved postoperative inflammatory markers and hepatic enzymes.^21^

Postoperative pain experienced after the application of pneumoperitoneum in minimally invasive gastrointestinal surgery can be attributed to several distinct sources, each contributing to varying degrees.^50,51^ These pain sources encompass visceral pain within the abdominal cavity, parietal pain related to incisions, and referred visceral pain manifesting as shoulder discomfort. The underlying mechanism behind referred visceral pain can be traced to the persistent stretching of the peritoneum during the insufflation process necessary to establish pneumoperitoneum. This continual stretching induces discomfort that can radiate to distant areas, such as the shoulder, creating referred pain sensations. Additional contributors to postpneumoperitoneum pain include the stretching of the peritoneal lining itself, irritation of the diaphragm, potential diaphragmatic injuries, and the positioning of the shoulder during surgery. These factors collectively contribute to the complex interplay of pain experiences in patients undergoing minimally invasive gastrointestinal procedures. Understanding these various sources of discomfort is vital for effectively managing and mitigating pain in patients undergoing surgery with pneumoperitoneum.^51,52^ Hypothetically, decreased insufflation pressures should reduce the amount of visceral pain experienced by the patient. However, this issue remains controversial.

On the other hand, this review has identified reports that low-pressure pneumoperitoneum can negatively affect surgical access, surgeon comfort, and surgeons’ assessment of visibility, which is an important consideration. This is important considering how ergonomics can impact the efficiency, safety, and comfort for the operating team.^53^ Albers et al^21^ also reported that 25% of patients required the pressure to be increased to obtain better visualization. Poor visibility can potentially lead to a greater chance of iatrogenic injury to surrounding structures, longer operating times, or conversion to open.^53,54^ Low pressure pneumoperitoneum was only associated in 1 study with significantly longer operating times, although the frequency of reporting for operating times was variable.^28^ Again, these results are susceptible to the inherent limitations already described and should be interpreted with caution. However, these considerations should be made in future studies.

The present study is limited because of the high variability and high risk of bias identified between studies. This variability is also seen in reported outcomes, which included a combination of abdominal pain, shoulder tip pain, pain on forced cough, or overall pain. There was also variability in the control group pressures ranging from 12 to 18 mm Hg and the intervention pressure from 5 to 12 mm Hg. VAS pain scores were reported at different times postoperatively making meaningful comparisons difficult. Reporting of neuromuscular blockade was not standardized and there were differences in the number of ports used 3 to 5 and intra-abdominal access techniques (Veress vs. open Hassan technique). Analgesic regimes varied substantially as did the use of local anesthetic. The predominance of studies in LC meant the ability of this SR to assess the benefits of low-pressure pneumoperitoneum in gastrointestinal surgery is limited. Consequently, this also means there is a higher proportion of females reported in the literature.

We would recommend that future studies assessing low-pressure pneumoperitoneum should be performed with a validated outcome such as the QoR-40 questionnaire. We strongly recommend a more standardized reporting to allow for better assessment in the future. One important aspect is to standardize the values for low-pressure pneumoperitoneum. Our suggestion would be that >12 mm Hg is standard pressure, 8 to 12 mm Hg is low pressure, and <8 mm Hg is ultra-low pressure. Future studies must also be more uniform in reporting a number of ports used, local anesthetic, neuromuscular blockage, perioperative analgesic regimes, and baseline demographic data.

In conclusion, this systematic review sheds light on the complexities surrounding the use of low-pressure pneumoperitoneum in gastrointestinal surgery. While the majority of the included studies focused on laparoscopic cholecystectomy (LC), we encountered a substantial degree of variability in reported outcomes. It is evident that this area of research requires more rigorous and standardized approaches to generate meaningful insights. Our study’s findings prompt a nuanced reflection on the available evidence, emphasizing the need to interpret aggregated results cautiously. Currently, there is insufficient evidence to suggest that low-pressure pneumoperitoneum is beneficial for patients undergoing minimally invasive gastrointestinal surgery.
